# Notes and News

**Published:** 1903-07-25

**Authors:** 


### Notes and News.

A successful meeting of the Marylebone District of the League of Mercy was held by Lord and Lady Farquhar on Saturday at 7 Grosvenor Square. Excellent progress in the district was reported.

Sir William Broadbent laid the foundation stone of the Passmore Edwards' Administrative House at the Colony for Epileptics at Chalfont St. Peter, on Monday. The company present visited the homes and buildings of the colony, and an exhibition of the work of the colonists was on view in the recreation hall.

The question of the transmission of tuberculosis to human beings by animals and *vice versa* continues to be a cause of dispute in medical circles. This was very forcibly shown at a recent meeting of the Berlin Medical Society, when a paper was read by Dr. Kossel, of the Imperial Health Department, repudiating as the result of experiment the communicability of the contagion. This paper was followed by one by Professor Orth, Virchow's successor at Berlin, quoting instances of tuberculosis in calves from bacilli taken from human beings.

Probably one of the most difficult of social problems is that of the unskilled labourer, who, without definite vocation or identification with any particular trade, has no ambition or prospect of betterment, and is seldom permanently employed. An important step towards the solution of the difficulty has been introduced by Messrs. Scrubb and Company, the manufacturers of the well-known and excellent cleansing preparation "Scrubb's Ammonia." This firm employ unskilled lads and give them encouragement and practical opportunity of learning the various trades that are in operation at their works, which include carpentering and other skilled labour. Certificates, medals, and bonuses are awarded for diligence, punctuality, and long service, and many of the boys leave their bonuses with the firm to accumulate at the rate of 5 per cent.

Prizes and certificates were presented to the successful students of the London Hospital Medical College on Monday by Mr. W. Dowis Hoare, chairman of the College Board.

The late Miss M. P. Leggatt, of Bramford, Suffolk, has bequeathed £2,000 to the East Suffolk and Ipswich Hospital to found and endow two beds in memoriam of her family, and £1,000 to the Royal Berkshire Hospital.

We are asked to state that full particulars of the eleventh International Congress of Hygiene and Demography, to be held in Brussels from 2nd to 8th September, 1903, with the travelling and hotel arrangements, may be obtained from Dr. Paul F. Moline, Hon. Sec. British Committee, 42 Walton Street, Chelsea, S.W.

The memorial scheme to the late Emperor and Empress Frederick, for London, is gradually progressing. The scheme provides for the endowment of a memorial bed in the new Hospital for Women for patients suffering from cancer, at a cost of £2,000. About £700 has been contributed, and further contributions may be sent direct to the bankers, Messrs. Barclay & Co., 1 Pall Mall East, for the Emperor and Empress Frederick Memorial.

A Convalescent Home has been opened at Radcliffe, called the Bealey Memorial Convalescent Hospital. It has been erected by Mr. A. C. Bealey in memory of his father and mother. There are two wards for four patients in each, these wards forming wings to the main administration building of two storeys in the centre. Mr. Bealey, besides paying for the establishment of the hospital, has also endowed it. Mr. James Sellers is the architect.

An interesting presentation has been made to Countess Howe by the officers and men of the Imperial Yeomanry as an expression of their gratitude and esteem for her services as president of the I. Y. Hospital Committee during the South African War. The gift took the form of an equestrian group modelled in solid silver, and represented a mounted yeoman reining in his horse. The subject is admirably chosen, and the work has been excellently executed by Messrs. Elkington and Co.

The fifth annual French medical tour to the health resorts in France will start from Toulouse this year, and conclude at Lyons. The programme includes visits to Salies-du-Salat, Aulus, Ax-les-Thermes, Ussat, Les Escaldes, Font Rosneu, Mont Louis, Carcanières, Alet, Molitg, Le Vernet, Amélie-les-Bains, La Preste, Le Boulou, Banyuls-sur-Mer, Lamalou, Montmirail, Vals, and Le Mont Pilat. The price of the tour between Toulouse and Lyons is about £14, inclusive of all expenses. For ladies accompanying their husbands £16. The country to be traversed is interesting medically and historically, and much of it is very beautiful. A good deal of the travelling is accomplished in carriages. The time occupied is from September 10 to 23. All particulars can be obtained from Dr. Carron de la Carriere, 2 Rue Lincoln, Paris.

